# Exciton–Polariton Valley Hall Effect in Monolayer Semiconductors on Plasmonic Metasurface

**DOI:** 10.1021/acsphotonics.4c01554

**Published:** 2025-03-04

**Authors:** Chien-Ju Lee, Hsin-Che Pan, Fatemeh HadavandMirzaee, Li-Syuan Lu, Fei Cheng, Tsing-Hua Her, Chih-Kang Shih, Wen-Hao Chang

**Affiliations:** † Department of Electrophysics, National Yang Ming Chiao Tung University, Hsinchu 30010, Taiwan; ‡ Department of Physics and Optical Science, The University of North Carolina at Charlotte, Charlotte, North Carolina 28223, United States; § Department of Physics, The University of Texas at Austin, Austin, Texas 78712, United States; ∥ Research Center for Applied Sciences, Academia Sinica, Taipei 11529, Taiwan

**Keywords:** 2D materials, Transition metal dichalcogenides, MoSe_2_, Valley degree of freedom, Exciton−polariton valley Hall effect

## Abstract

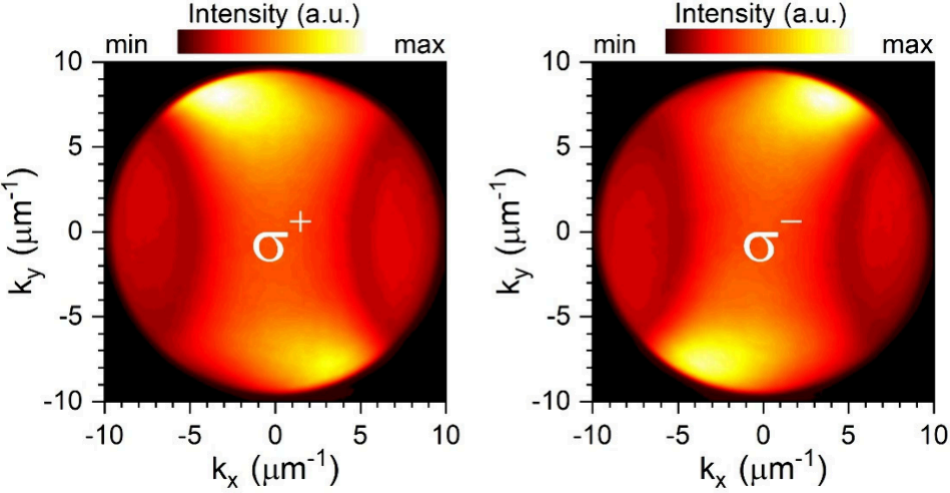

Excitons in monolayer transition metal dichalcogenides (TMDs) possess the valley degree of freedom (DOF), which is regarded as a pseudospin (in addition to charge and spin DOF) and can be addressed optically by using polarized light. Incorporating monolayer TMDs into an optical microcavity in the strong coupling regime further enables the formation of valley polaritons that are half-light and half-matter quasiparticles with addressable spin and momentum through the spin–orbit interactions of light, in analogy with the spin-Hall effect in electronic systems. By placing monolayer TMDs on a plasmonic metasurface to enable strong coupling between excitons and surface plasmon polaritons (SPPs), we report here the observation of valley resolved polaritons in momentum space and a large separation in real space. The directional coupling of valley polaritons originated from the intrinsic spin-momentum locking associated with SPPs, resembling a photonic version of the valley Hall effect for polaritons. The spatially routed valley polaritons provide a unique pathway for transporting and detecting the valley DOF through circular polarization of light for valleytronic applications.

## Introduction

**Figure 1:**
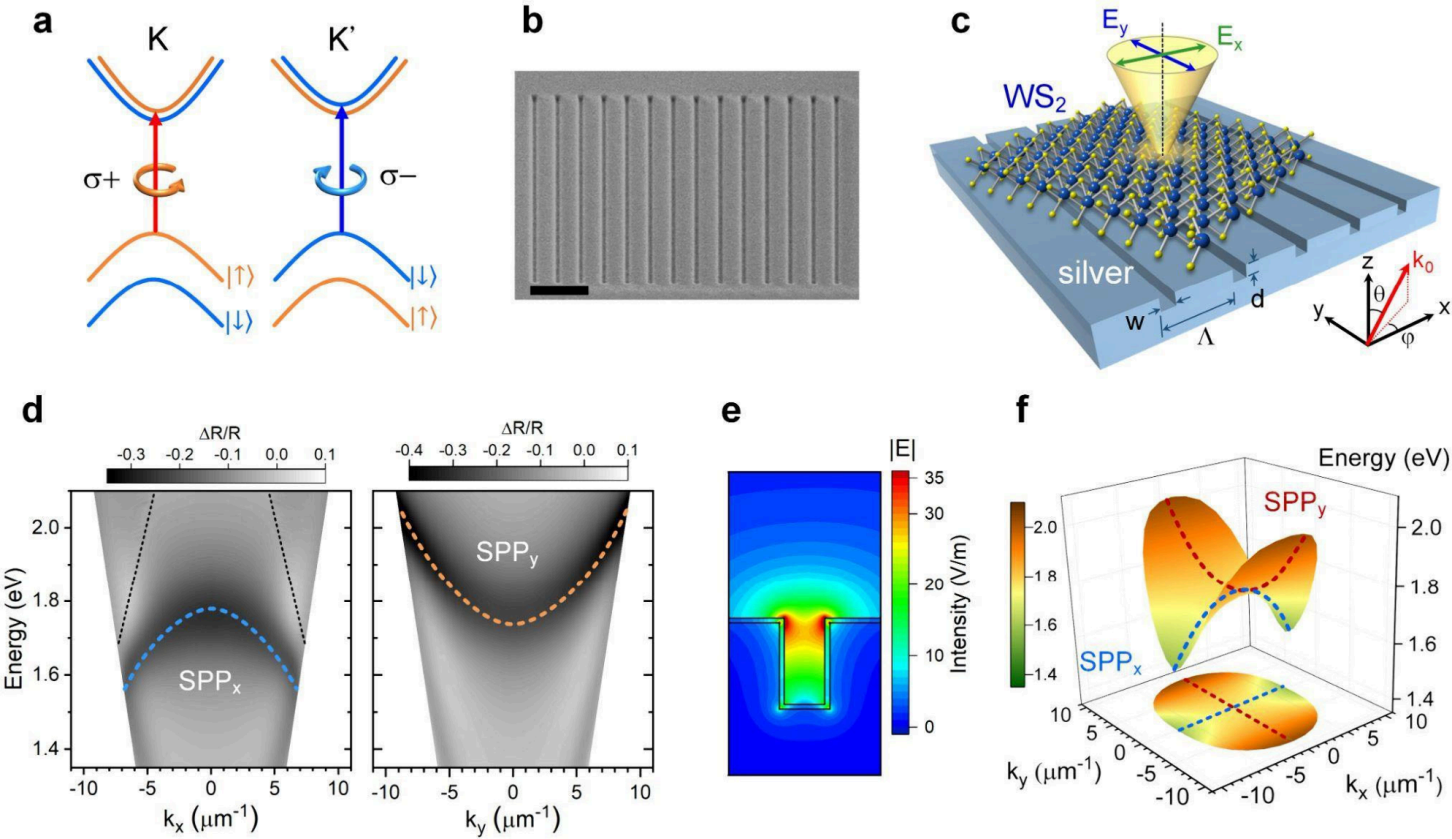
Monolayer WS_2_ on a nanogroove array with anisotropic SPP dispersions. (a) Schematic of the optical selection rules of excitons in monolayer WS_2_ at the K and K′ valley with σ^+^ and σ^–^ circular polarizations. (b) Scanning electron microscopy image of a nanogroove array. The scale bar is 1 μm. (c) Schematic of a 2D semiconductor on top of a plasmonic metasurface consisting of a linear array of nanogrooves with depth *d*, width *w*, and period Λ fabricated on a single-crystalline silver film. (d) The dispersion of SPPs along *k*_*x*_ (left) and *k*_*y*_ (right) of a nanogroove array with *w* = 50 nm, *d* = 70 nm, and Λ = 400 nm calculated by FDTD. The two linear dispersions (dotted lines) are the diffractions grazing to the metal surface (Rayleigh anomalies). (e) Electric field distribution of the guided mode in the groove. (f) A 2D band dispersion of the nanogroove array in (d) constructed from FDTD calculations.

Monolayer transition metal dichalcogenides (TMDs) exhibit a helicity-dependent optical selection rule at the K and K′ valleys^1−5^ (Figure 1a). As a result, excitons in different valleys can be selectively addressed by σ^+^ and σ^–^ circularly polarized light. The K and K′ valleys further exhibit opposite Berry curvatures,^6^ which can lead to a valley-dependent spatial separation of Bloch electrons or excitons known as the valley Hall effect.^2,7−9^ The valley Hall effect for Bloch electrons has been demonstrated in MoS_2_ monolayers^10^ and bilayers.^11^ The excitonic valley Hall effect has also been realized in monolayer MoS_2_^12^ and MoS_2_/WSe_2_ heterobilayers.^13^ However, practical implementation of exciton-based valleytronics applications is still hindered by the rapid depolarization of valley pseudospin and short propagation lengths for excitons.

Engineering the optical environment of TMDs can provide additional tuning knobs for controlling the polarization and propagation of the valley pseudospin. This has been achieved by incorporating monolayer TMDs into an optical microcavity, where polaritons that are half-light and half-matter quasiparticles can be formed when excitons and the cavity photons are strongly coupled.^14−20^ The polaritons not only preserve the valley pseudospin of excitons in TMDs that can be addressed by circularly polarized light but also significantly enhance the valley polarization due to the cavity-modified exciton dynamics.^15−18^ With longer lifetimes and enhanced valley polarizations, exciton–polaritons can provide a promising alternative for manipulating the valley degree of freedom (DOF).^19,20^

In addition, photons also carry both spin (circular polarization) and orbital angular momentum. Due to the spin–orbit interactions of light,^21−23^ the propagation direction (the wavevector or momentum) of photons becomes controllable through harnessing their circular polarization. Coupling TMDs to photonic or plasmonic nanostructures thus enables a spatial separation of valley pseudospin through spin–orbit coupling.^24−27^ Directional coupling of circularly polarized light has been demonstrated experimentally in various photonic structures, including metal surfaces,^28−32^ nanofibers,^33,34^ and various waveguides.^35−37^ The polarization directionality arises from the unique transverse spin angular momentum (t-SAM) of a surface propagating evanescent wave that is unidirectionally locked with the direction of propagation, i.e., the so-called spin-momentum locking.^21,22^ In this context, when the valley excitons and surface waves, such as surface plasmon polaritons (SPPs) at a metal–vacuum interface,^38,39^ are strongly coupled, the valley polaritons that possess the character of surface waves can be routed into different paths, a phenomenon referred to as polariton valley Hall effect.

Here, we demonstrate the polariton valley Hall effect in monolayer WS_2_ on a plasmonic metasurface formed by a one-dimensional array of metallic subwavelength grooves (Figure 1b). Fourier-space spectroscopy was used to measure the dispersions of the exciton–polaritons. Strong coupling between valley excitons and SPP modes of the nanogroove arrays has been achieved and demonstrated through anti-crossing in their dispersions. We also performed polarization-resolved photoluminescence (PL) imaging and observed a valley-contrasting propagation of valley polaritons in both real and momentum space (see Supporting Information 1 and 3 for experimental details). The spatially routed valley polaritons provide a unique pathway for detecting the valley degree of freedom for valleytronics applications.

## Results and Discussion

The nanogroove arrays were fabricated on an epitaxially grown single-crystalline silver film^40^ using focused ion beam (FIB) milling. The use of single-crystalline silver film grown by molecular beam epitaxy ensures a long SPP propagation length with low optical loss, which is critical for reaching the strong light–matter coupling regime. A 5 nm-thick Al_2_O_3_ was deposited on the silver film by atomic layer deposition to protect the silver from oxidation. Monolayer WS_2_ grown by chemical vapor deposition was transferred onto the nanogrooves as depicted in Figure 1c. The thin Al_2_O_3_ layer can also eliminate the radiation quenching caused by the charge transfer between WS_2_ and silver.^41−43^

We designed the structure of the nanogroove arrays by finite-difference time domain (FDTD) simulations. The SPP resonances depend on the width (*w*), depth (*d*), and period (Λ) of the nanogrooves. Calculations suggest that nanogrooves with *w* = 40–60 nm, *d* = 40–85 nm, and Λ = 400–600 nm yield SPP modes near the exciton resonance of monolayer WS_2_. Choosing nanogrooves with shallow depth and small width (i.e., in the weak perturbation regime) can minimize SPP radiative damping, resulting in a narrower SPP spectral width to facilitate reaching the strong coupling regime.^38,44,45^ The nanogroove array can support SPP modes propagating either perpendicular (SPP_*x*_) or parallel (SPP_*y*_) to the groove direction (*y*) (Supporting Information 2). Figure 1d shows the calculated dispersion relations by FDTD for SPP_*x*_ and SPP_*y*_ of a bare nanogroove array. For SPP_*x*_, the periodic structure folds the dispersion of SPP on the flat surface back into the light cone and forms plasmonic band gaps at the Brillouin zone edges.^46^ On the other hand, SPP_*y*_’s are guided modes (Figure 1e) along the grooves (also known as channel polaritons^47,48^) and behave as coupled waveguide modes.^49−51^ Both SPP_*x*_ and SPP_*y*_ can couple to light at certain angles θ when the in-plane momentum of light *k*_0_sin θ matches the SPP wavevector *k*_SPP_(52,53) (Supporting Information 1, eq S1). Figure 1f shows the two-dimensional (2D) band dispersion constructed by combining the dispersion relations along the *x*- and *y*-directions. The 2D dispersion suggests that the SPP is propagating anisotropically in the *x*–*y* plane.

**Figure 2:**
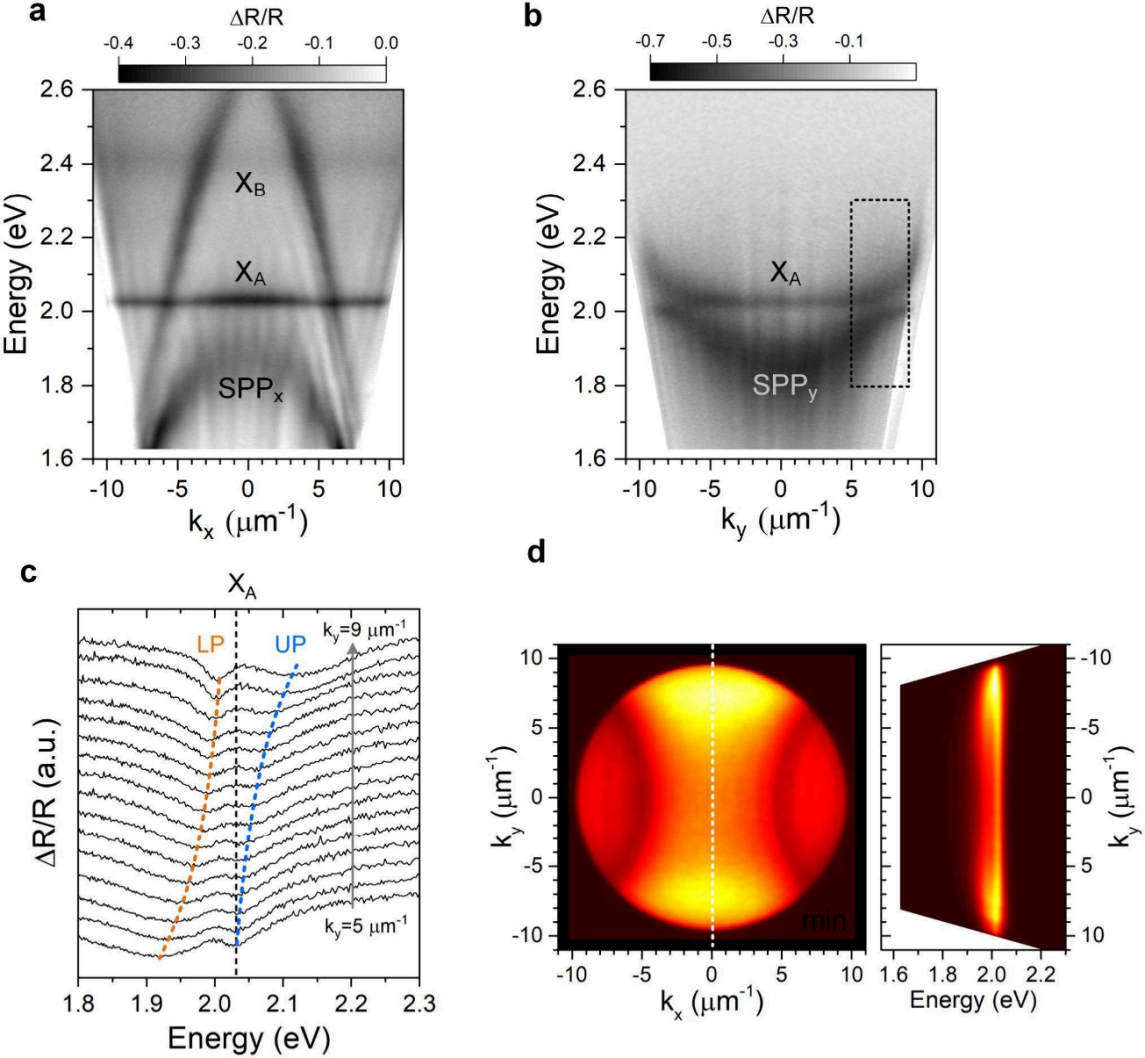
Strong coupling between excitons in WS_2_ and SPPs of nanogroove arrays. (a, b) The dispersions of a coupled WS_2_-nanogroove array along *k*_*x*_ (a) and *k*_*y*_ (b) obtained from angle-resolved reflectivity measurements. An anti-crossing between the SPP_*y*_ and WS_2_ A-exciton is observed in (b). The two linear dispersions in (a) are the diffractions grazing the metal surface (Rayleigh anomalies). (c) The angle-resolved reflectivity spectra near the anti-crossing region marked in (b). (d) The k-space PL image and PL dispersion. The left panel shows the unpolarized k-space PL image. The right panel shows the PL dispersion along the *y*-direction, indicated by the vertical dashed line in the central panel. The dimensions (*w*, *d*, Λ) of the nanogroove array are 50, 80, and 400 nm.

We performed angle-resolved reflectivity measurements to characterize the dispersions of WS_2_ exciton and the SPP modes (Supporting Information 1 and 3). Figure 2a,b shows the measured dispersions of a representative sample (with nominal values of *w* = 50 nm, *d* = 80 nm, and Λ = 400 nm) along *x* -and *y*-directions, respectively. The A-exciton (X_A_) and B-exciton (X_B_) absorptions in monolayer WS_2_ are observed at 2.03 and 2.4 eV, respectively. In the *x*-direction, the resonant energy of SPP_*x*_ is lower than that of the X_A_ resonance (Figure 2a). On the other hand, SPP_*y*_ resonates to X_A_ at *k*_*y*_ ≈ ± 7 μm^–1^ (Figure 2b). An anti-crossing between SPP_*y*_ and X_A_ can be observed, manifesting the formation of an exciton-SPP polariton in the strong coupling regime. Figure 2c displays the reflectivity spectra near the anti-crossing. We used a coupled oscillator model^14,38^ (Supporting Information 4) to analyze the anti-crossing. The dispersion relations of the upper polariton (UP) and lower polariton (LP) branches of the hybrid exciton–polariton modes are given by
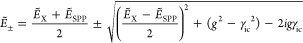
1where *Ẽ*_+_ and *Ẽ*_–_ are the complex resonance energies of UP and LP branches, respectively; *Ẽ*_X_ = *E*_X_ – *i*Γ_X_/2 and *Ẽ*_SPP_ = *E*_SPP_ – *i*Γ_SPP_/2 are the complex energies of the uncoupled monolayer WS_2_ excitons and SPPs, respectively; *g* is the coherent coupling strength, and *γ*_*ic*_ is the incoherent coupling strength.^38^

By fitting the angle-dependent resonant energies and line widths of UP and LP branches to the real and imaginary part of eq 1, we deduced a coherent coupling strength of *g* = 59 ± 2 meV and an incoherent coupling strength of *γ*_*ic*_ = 5 ± 1 meV. For the fitted X_A_ line width Γ_X_ = 39 meV and the SPP line width Γ_SPP_ = 195 meV, the coupling strength *g* marginally satisfies the criterion for strong coupling, i.e., *g* ≳ (Γ_X_ + Γ_SPP_)/4. The smaller γ_ic_ indicates that the exciton-SPP coupling is dominated by the coherent coupling through Rabi oscillations but with a minor contribution from an incoherent coupling pathway,^38^ which may be caused by the spontaneous emissions from excitons and SPPs into the surrounding and then be reabsorbed by each other (Supporting Information 4). Additional data for the coupling between monolayer WS_2_ and other nanogroove arrays are shown in Supporting Information 5.

To characterize the polariton emission properties, we measured the photoluminescence (PL) intensity distribution in the momentum space, i.e., the k-space PL imaging, by projecting the emission patterns onto the Fourier plane (Supporting Information 3). Figure 2d shows the k-space PL image without polarization selection using a linearly polarized 532 nm laser as the excitation source. The k-space PL image exhibits two bright lobes centered at *k*_*x*_ = 0 and *k*_*y*_ ∼ ±7 μm^–1^. Compared with the PL dispersion along the *y*-direction (right panel in Figure 2d), the two bright regions in the k-space PL image correspond to the emissions from LP populated in the vicinity of zero detuning. The polariton formation processes can be understood as follows.^14,32^ The nonresonant photoexcitations first create X_A_ excitons with high in-plane wavevectors in WS_2_. After energy-momentum relaxations, the excitons are strongly coupled to the nanogroove waveguide mode (SPP_*y*_), resulting in the formation of X_A_-SPP_*y*_ polaritons with nonzero in-plane momentum propagating predominantly along the nanogrooves. The incoherent coupling between X_A_ and SPPs can induce angle-independent X_A_ emissions, which contributes as a relatively weaker emission background in the k-space PL image.

**Figure 3:**
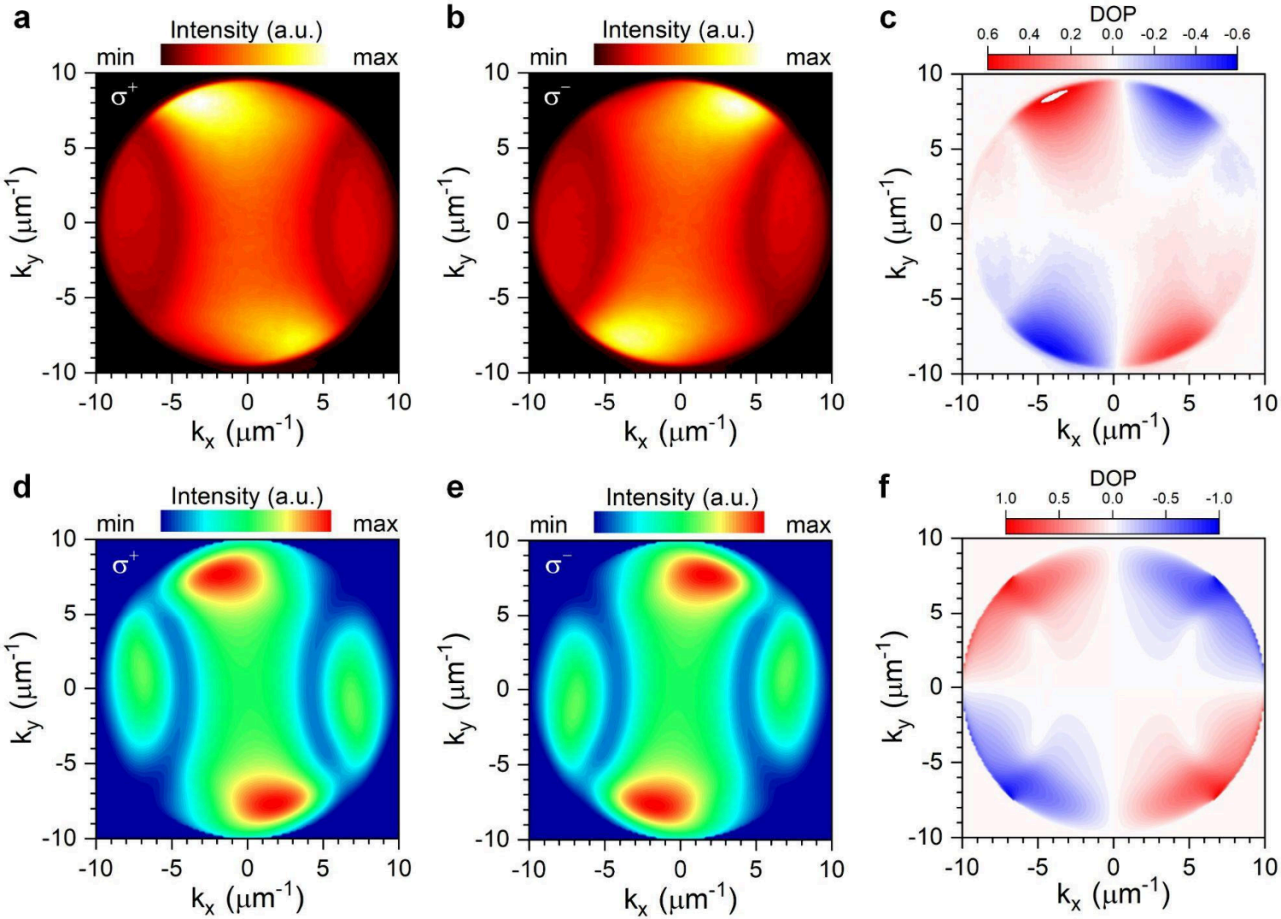
Far-field separation of valley polariton emission in k-space. (a, b) The k-space imaging of σ^+^ (a) and σ^–^ (b) PL from WS_2_ on nanogrooves. (c) The valley polarization contrast (degree of circular polarization) in k-space extracted from (a, b). (d–f) Simulated far-field distributions induced by σ^+^ (d) and σ^–^ (e) ^–^ dipoles and the corresponding degree of circular polarization (f).

We investigate the propagation of valley polaritons by polarization-resolved k-space PL imaging by using a combination of polarizers and waveplates in the detection path (see Supporting Information 1 and 3 and Figure S6 for a detailed description of the optical setup and experimental procedures). Figure 3a,b shows the k-space PL images with σ+ and σ– circular polarizations, respectively. A separated emissions of σ^+^ and σ^–^ polaritons in momentum space can be observed. Since the excitation light is linearly polarized, the created valley excitons and hence the valley polaritons formed subsequently are expected to have equal populations at both K and K′ valleys. The separation of σ^+^ and σ^–^ polaritons indicates that the polaritons at the K and K′ valleys are routed to opposite directions, unambiguously demonstrating the occurrence of the polariton valley Hall effect.^54,55^ We have evaluated the degree of circular polarization defined as (*I*_σ+_ – *I*_σ–_)/(*I*_σ+_ + *I*_σ–_), where *I*_σ+_ (*I*_σ–_) is the σ^+^ (σ^–^) component of PL intensity to quantify the valley polarization. The resultant valley polarization contrast in the k-space is shown in Figure 3c. A maximum degree of valley polarization of up to ∼0.6 has been reached, which is higher than the polarization contrasts observed in platforms based on hyperbolic metamaterial^27^ (∼0.2) and plasmonic hole arrays^26^ (∼0.4).

**Figure 4:**
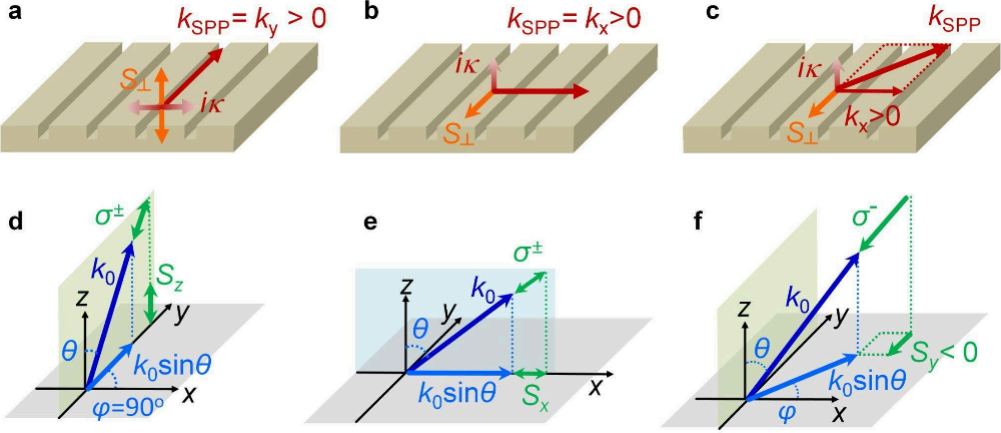
The t-SAM of SPP and the spin projection of far-field radiation. (a–c) The transverse spin angular momentum *S*_⊥_ of SPP propagating along the *y*-direction (a), *x*-direction (b), and oblique direction (c). (d–f) The corresponding longitudinal spin component of exciton far-field radiation (σ^±^ polarizations) projected on the *x*–*y* plane.

The physical origin of the polariton valley Hall effect can be understood from the plasmonic spin-Hall effect^51^ on the nanogroove array. The valley exciton can be considered as an in-plane circular dipole with σ^±^ polarizations. The near-field coupling between the exciton dipoles and the SPP leads to the formation of exciton-SPP polaritons that can propagate along the nanogrooves (the guided SPP_*y*_ in the *y*-direction) or perpendicular to the nanogrooves (the surface confined SPP_*x*_ in the *x*-direction). The wavevector of SPP can be expressed as *k*_SPP_ = *k*_SPP_^∥^ρ̂ + *i*κ*n̂*, where  is the in-plane wavevector of SPP; ρ̂ and *n̂* are unit vectors in the in-plane and surface normal directions, respectively The formation of exciton-SPP polariton requires a momentum matching condition between the wavevectors of SPP and exciton far-field radiation (*k*_0_), i.e., *k*_*SPP*_^∥^ = *k*_0_sin θ, where θ is the polar angle of *k*_0_ (Figure 4). The directional coupling of valley polaritons, on the other hand, requires additional matching conditions between the t-SAM *S*_⊥_ of SPP and the longitudinal spin component of exciton far-field radiation (σ^±^ polarizations) projected on the *x*–*y* plane, as illustrated in Figure 4. The t-SAM of SPP can be expressed as *S*_⊥_ = (*k*_*SPP*_^∥^ρ̂ × κ*n̂*)/(*k*_*SPP*_^∥^)^2^.^21,22^ We first consider the case of guided SPP_*y*_ with *k*_*x*_ = 0 (Figure 4a,d). The guided SPP_*y*_ propagating along +*y*-direction supported by the nanogrooves with symmetric sidewalls can carry t-SAM *S*_⊥_ pointing along +*z* and −*z* directions, which can couple to excitons at both K and K′ valleys with σ^+^ and σ^–^ polarizations.^56^ Therefore, no directional coupling of σ^+^ and σ^–^ polarizations in the *y*-direction is expected, corresponding to the zero polarization contrast at *k*_*x*_ ≈ 0 observed in Figure 3c. For the case of SPP_*x*_, the evanescent waves confined at the metal surface carries a t-SAM *S*_⊥_ along −*y* (+*y*) for *k*_*x*_ > 0 (*k*_*x*_ < 0) due to the spin-momentum locking (Figure 4b,e). However, the spin projection of exciton far-field radiation has no spin component along the *y*-direction (*S*_*y*_ = 0), which results in no directional coupling in the *x* direction. Now, we consider polaritons propagating obliquely with a nonzero *k*_*x*_ and *k*_*y*_ (Figure 4c,f). For SPP with *k*_*y*_ > 0 and *k*_*x*_ ≠ 0, the spin-momentum locking for SPP*x* dictates a directional coupling between the SPP with *k*_*x*_ > 0 (*k*_*x*_ < 0) and the far-field radiation with σ^–^ (σ^+^) polarization that exhibits a spin component *S*_*y*_ < 0 (*S*_*y*_ > 0) when projecting on the *x*–*y* plane (Figure 4c,f). For the case with *k*_*y*_ < 0, the circular polarization of the preferentially coupled far-field radiation reverses, leading to a quadrant distribution of σ^–^ and σ^+^ and far-field radiation patterns observed in Figure 3a,b. To further confirm our assertion, we carried out FDTD calculations to simulate the far-field patterns of the nanogroove array (Supporting Information 5). In the simulations, an in-plane circular dipole was placed on the nanogroove array to excite the SPP on the *x*–*y* plane. The resulting far-field radiation patterns excited by the σ^+^ and σ^–^ dipoles, as well as the degree of valley polarization, are shown in Figure 3d,f. The simulations reproduce our experimental observations very well, demonstrating separated σ^+^ and σ^–^ radiations in momentum space.

**Figure 5:**
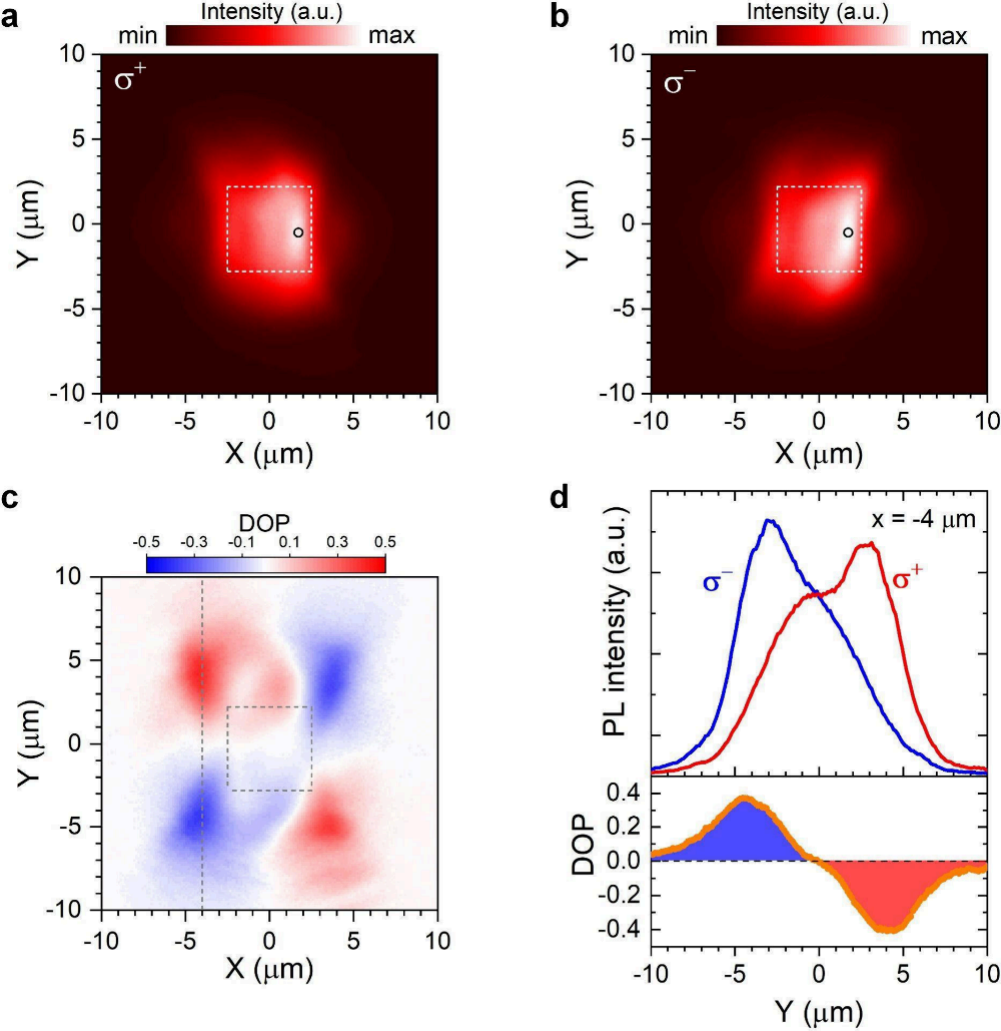
Routing of valley polaritons in real space. (a, b) Real space PL images of σ^+^ (a) and σ^–^ (b) components of polariton emissions from the monolayer WS_2_ on nanogrooves. The dashed box indicates the region of the nanogroove array. The circle marks the position of the laser excitation. We intentionally shifted the excitation position toward the right side of the array in order to see polariton propagation toward the left side of the array. (c) Real space distribution of the valley polarization contrast extracted from (a) and (b). (d) A line scan of PL intensity distribution of the σ^+^ and σ^–^ components and the corresponding degree of circular polarization extracted from the dashed line at *x* = −4 μm indicated in (c).

The directional coupling induced by spin-momentum locking of SPP can lead to valley-contrasting polariton propagations not only in k-space but also in real space. We have measured the real space polarization-resolved PL images and quantified the valley polarization by the degree of circular polarization (Supporting Information 1B). As shown in Figure 5a,b, the σ^+^ and σ^–^ PL images exhibit asymmetric shapes and deviate from the image taken without polarization selection (Figure S12c). Both of the σ^+^ and σ^–^ PL images show elongated shape along different diagonals of the nanogroove array (indicated by the white dashed squares). This can be attributed to the anisotropic transports of valley polaritons in real space guided by the nanogroove array. Figure 5c shows the spatial valley polarization by calculating (*I*_σ+_ – *I*_σ–_)/(*I*_σ+_ + *I*_σ–_) from the measured PL intensities shown in Figure 5a,b. The highest degree of valley polarization reaches ∼0.4 at around *x̃* ± 4 μm from the center of the array. The spatial profiles of PL intensity and the polarization contrast at *x* = −4 μm (dashed line in Figure 5c) are shown in Figure 5d. The σ^+^ and σ^–^ PL peaks can be separated up to ∼6 μm apart.

We noted that the lateral extent of PL emission has expanded beyond the boundary of the nanogroove array and is significantly larger than the exciton diffusion length in monolayer TMDs, typically on the order of hundreds of nanometers and limited to a few micrometers under very high excitation power.^57−61^ By measuring the intensity profile of PL from WS_2_ on sapphire, we estimated the exciton diffusion length *L*_D_ in monolayer WS_2_ to be about *L*_D_ ∼ 0.2 μm (Figure S12a). For WS_2_ on flat Ag surface, the measured PL spot size corresponds to a larger diffusion length *L*_D_ ∼ 3.0 μm due to the propagation of SPPs excited and reabsorbed by the excitons (Figure S12b). However, this process is incoherent and cannot preserve the phase relationship associated with valley excitons. When monolayer WS_2_ is placed on top of a nanogroove array, the strongly coupled excitons and SPPs dramatically change the transport behavior, as can be seen from the very different spatial images of PL from WS_2_ on nanogrooves and that on flat Ag (Figure S12b,c). The dramatically changed transport behavior can be explained by the anisotropic in-plane momentum of the valley polaritons with a large *k*_*y*_ ∼ ±7 μm^–1^ with a *k*_*x*_ ∼ 0, where the polariton exhibits a large group velocity (*v*_g_ = dω/d*k*_*y*_) along the *y*-direction (Figure S13), leading to an elongated PL distribution in the *y*-direction. Considering a group velocity of about 30–50 μm/ps for the LP at zero detuning and a SPP lifetime of ∼100 fs,^45,62^ the polariton diffusion length is expected to be ∼3–5 μm, which is in agreement with the measured *L*_D_ ∼ 4.2 μm for the polariton along the *y*-direction. As the polaritons transport across the boundary of the nanogroove array, the polaritons with a large in-plane momentum are converted into SPPs on the flat silver surface with well-defined transverse spin angular momentum, which preserves the circular polarization of out-coupled photons by surface scatterers. This explains the observation of a high degree of circular polarization, even outside the nanogroove array (Figure 5c,d).

## Conclusion

In summary, we investigated the coherent interaction between excitons in the WS2 and SPPs supported by a plasmonic metasurface. Strong coupling between excitons and SPPs were demonstrated through the anti-crossing in their dispersions. Furthermore, the polarization-resolved momentum- and real-space PL images of the valley polariton exhibit higher degrees of circular polarization as compared to polariton emission reported in previous works.^15−18^ We attribute this to the combined effects of the strongly coupled exciton–plasmon polariton and plasmonic spin-Hall effect. The valley-contrasting propagation of valley polaritons in momentum space can be further improved by optimizing the design of subwavelength nanogroove arrays. The valley polaritons with opposite valley indices can be transported and separated further apart in real space by incorporating out-coupling structures to achieve an even higher valley polarization. Exploiting the negative refraction and hyperbolic dispersions offered by the metasurface can also open up exciting possibilities for manipulating valley polariton transports. Our results demonstrate a new pathway for transporting and detecting the valley degrees of freedom in 2D semiconductors, a crucial step toward implementing the valleytronics applications.

## Data Availability

All data needed to evaluate the conclusions in the paper are present in the paper and/or the Supporting Information. Additional data related to this paper are available from the corresponding authors upon reasonable request.

## Supporting Information Available

Methods, dispersion of SPP modes on a metallic nanogroove array, Fourier-space spectroscopy, coupled oscillator model, additional data for exciton-SPP coupling on other nanogroove arrays, numerical calculations, and propagation of valley polariton (PDF)
