# Supplementary material for: Exciton–Polariton Valley Hall Effect in Monolayer Semiconductors on Plasmonic Metasurface
Source: ACS Photonics. 2025 Mar 4;12(3):1351–8. doi: 10.1021/acsphotonics.4c01554 (PMC11926950; doi:10.1021/acsphotonics.4c01554)
Supplement: Supplementary file 1 — ph4c01554_si_001.pdf [file ph4c01554_si_001.pdf]

Supporting Information

Exciton-polariton valley Hall effect in monolayer semiconductors on plasmonic metasurface

Chien-Ju Lee[1], Hsin-Che Pan[1], Fatemeh HadavandMirzaee[2], Li-Syuan Lu[1], Fei Cheng[3], Tsing-Hua Her[2], Chih-Kang Shih[3], Wen-Hao Chang[1,4,*]

[1] Department of Electrophysics, National Yang Ming Chiao Tung University, Hsinchu 30010, Taiwan

[2] Department of Physics and Optical Science, The University of North Carolina at Charlotte, Charlotte, NC 28223, USA

[3] Department of Physics, The University of Texas at Austin, Austin, Texas 78712, USA

[4] Research Center for Applied Sciences, Academia Sinica, Taipei 11529, Taiwan

*whchang@nycu.edu.tw (W.H.C.)

Table of contents

1. **Methods:**

   A. Fabrication of monolayer WS_2 on nanogroove arrays

   B. Optical measurements

   C. Numerical Simulations

2. **Dispersion of SPP modes on a metallic nanogroove array**
3. **Fourier-space spectroscopy**
4. **Coupled oscillator model**
5. **Additional data for exciton-SPP coupling on other nanogroove arrays**
6. **Numerical calculations**
7. **Propagation of valley polariton**

1. Methods

A. Fabrication of monolayer WS_2 on nanogroove arrays

The plasmonic metasurfaces consist of arrays of periodically arranged nanogrooves fabricated on a 200-nm-thick single-crystalline silver film using focused ion beam milling. The silver films were grown epitaxially on silicon substrate by molecular beam epitaxy[33]. Scanning tunneling microscopy, atomic force microscopy and x-ray diffraction measurements have been used to confirm the high crystalline quality and atomic flat surface of the silver film, which can support ultra-low-loss SPP modes with a propagation length up to ~80 μm at 632 nm[33]. A 5-nm Al_2O_3 layer was deposited on top to protect the silver from oxidation and to reduce the absorption loss of SPPs. **Figure S1** shows a SEM image of the single-crystalline silver film with a nanogroove milled by FIB.

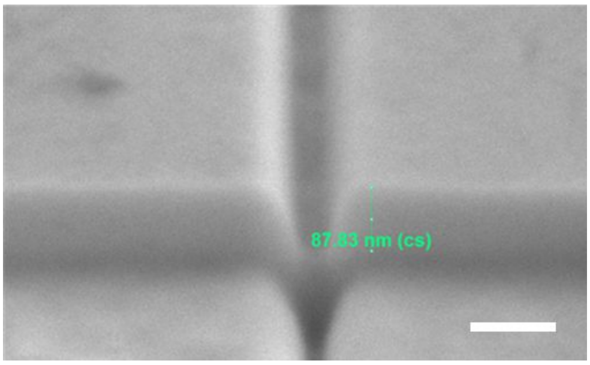

Figure S1 | A close-up SEM image of the silver film after focused ion beam milling. The surface and sidewall are smooth. The scale bar is 100 nm.

Monolayer WS_2 were grown on sapphire substrates by chemical vapor deposition. After growth, the monolayer WS_2 flakes were transferred onto the fabricated nanogroove arrays by the dry transfer method using a polydimethylsiloxane (PDMS) film. **Fig. S2** shows the optical microscope image of WS2 transferred onto nanogroove arrays. The nanogroove arrays were fabricated with groove width (w), depth (d) and period (Λ) in the range of $w =$ 40-60 nm, $d =$ 40-85 nm and $\Lambda =$ 400-600 nm, which yield dispersions covering the wavelength of exciton resonance at 610 nm for WS_2.

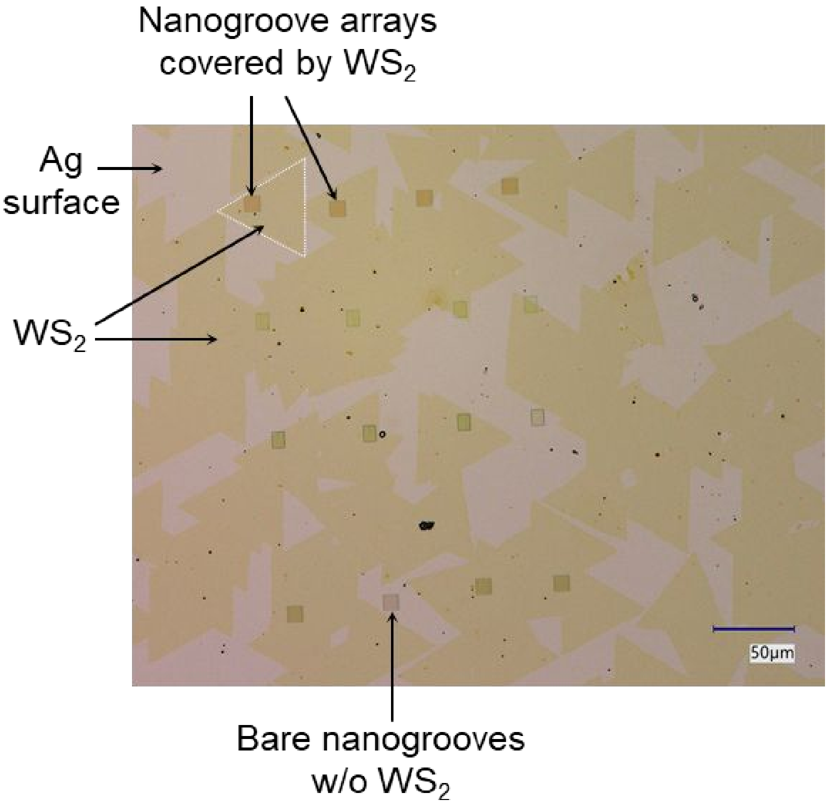

Figure S2 | Optical microscope image of nanogroove arrays covered with CVD-grown WS_2 flakes.

B. Optical measurements

The reflectivity and photoluminescence spectra of the TMD-nanogroove structures were measured by a home-built micro-photoluminescence (μ-PL) setup. Collimated white light from a broad band halogen lamp or a continuous wave laser with a wavelength of 532 nm was used for the reflectivity and PL measurements, respectively. A 100× objective lens (Olympus, NA = 0.95) was used to focus the excitation beam and collect the emission or reflection. The collected signals were then sent to a spectrometer equipped with an electron-multiplying CCD (EMCCDs) camera. For the reflectivity measurements, the reflected spectra from the WS2 flakes R_{2D} and from the substrate R_s were measured separately. The reflectivity spectra $\Delta R/R$ were determined by $\Delta R/R = (R_{2D} - R_s)/R_s$.

A Fourier-space optical system was constructed to obtain the dispersion relations and k-space PL images. A tube lens (L1 in Fig. S6) was placed at the position where its focal plane coincides with the back focal plane of the objective lens. The Fourier lens (L2) was positioned at a distance of f1 + f2 from L1 and focused light at the entrance of the spectrometer. The sample was mounted on a rotator so that the dispersions along different in-plane directions can

be measured by rotating the sample to match the orientation of k and the slit of the spectrometer. For real space measurements, L1 was removed. The real space PL images were obtained by directly imaging emission from the sample plane onto the spectrometer (see **Fig. S6**). By fully opening the entrance slit and rotating the grating of the spectrometer to zeroth order, the PL images can be captured by the CCD camera. For both the k-space and real space PL imaging, all the light signals from the sample plane were collected and filtered by a 550 nm longpass filter before sending to the spectrometer. The polarization of the photoluminescence was analyzed by a combination of a quarter-waveplate and a linear polarizer.

C. Numerical Simulations

Finite-difference time domain method (FDTD) was used to investigate the mode properties of the nanogroove arrays. The dispersion relations of SPP modes were calculated by monitoring the optical absorption of the nanogroove array using an incident broad-band light source from far-field and collecting the reflected light. A 5-nm of Al_2O_3 layer was added on the surface of nanogroove arrays conformally in all simulations. Periodic boundary conditions were used in both x- and y-directions. In the $\pm$ z-directions, perfectly matched layers (PML) were used. The 2D dispersion relation of a nanogroove array was calculated by changing the incident angle (θ and φ) of the light source. For instance, when calculating the dispersion of SPPx (SPPy), the incident angle of the light source was set to vary from $\theta = 0°$ to 70° with φ fixed at $\varphi = 0°$ ($\varphi = 90°$) (see Fig. 1c in main text). TM and TE waves were used to excite the SPPx and SPPy modes, respectively, where, in both cases, the electric fields are polarized along x-direction. The dielectric functions of Ag film and Al_2O_3 were taken from references [1] and [2], respectively.

For the calculation of reflectivity spectra with strong coupling to WS_2, a 0.7-nm dielectric layer was added on the Al_2O_3 surface to simulate the monolayer TMD. The real-part and imaginary-part of the dielectric function of monolayer WS_2 were extracted from experimentally measured reflectivity spectra of monolayer WS_2 on sapphire substrates by fitting the reflection spectrum to a superposition of multiple Lorentzian functions with an equal energy spacing of 3 meV and a constant linewidth of 10 meV [3].

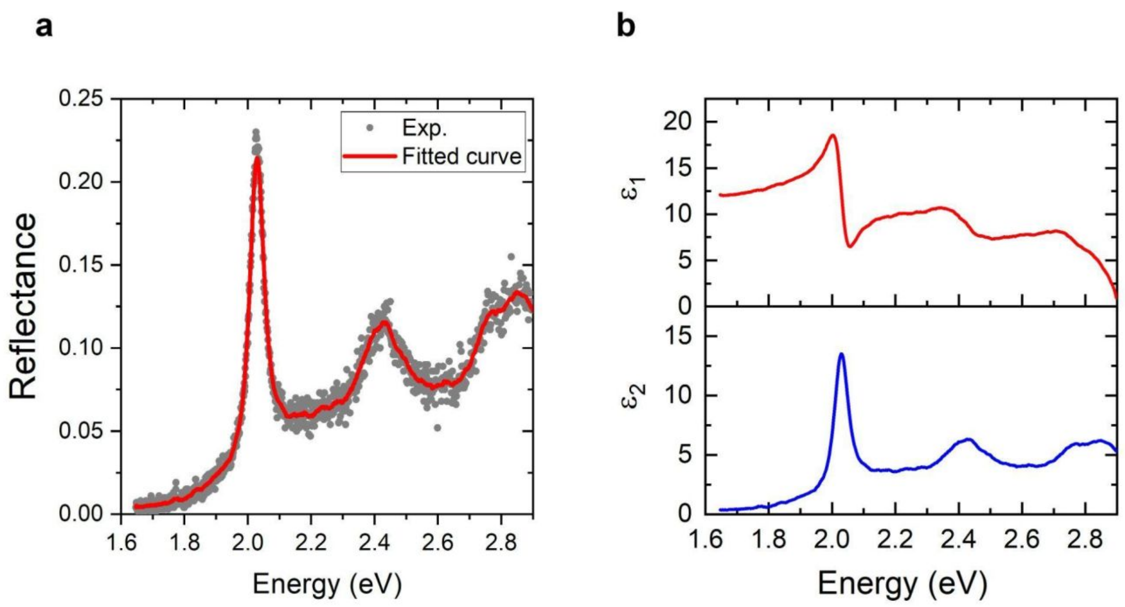

Figure S3 | a, fitting the reflection spectrum of monolayer WS2 to a superposition of multiple Lorentzian functions. **b,** The real and imaginary parts of the dielectric function deduced from the fitting.

The far –field polarization-resolved electric field distribution was calculated by placing in-plane circularly-polarized dipoles at the center of nanogroove arrays. The excited electric fields were monitored by a field detector on the metal surface. In the far-field calculation, perfectly matched layers were used in all the boundaries.

2. Dispersion of SPP modes on a metallic nanogroove array

The metallic nanogroove array exhibits anisotropic dispersions. The SPPs propagating perpendicular to the grooves (SPP_x) experience Bragg diffractions induced by the periodic structure[34]. **Fig. S4a** shows a schematic for the SPP dispersion along k_x modified by the periodic structure. The red line indicates the dispersion of SPP on flat metal surfaces. Due to the presence of periodically arranged grooves, the dispersion of SPP on the flat metal surface is folded back into the light cone by the Bragg diffractions and opens a band gap at Brillouin zone boundaries. Inside the light cone, the SPPs are unbound SPP modes and can couple to free-space light at a certain angle.

On the other hand, SPPs propagating parallel to the grooves (SPP_y) are dominated by the guided SPP modes confined in the nanogrooves known as channel polaritons. In principle, the

dispersion of guided SPP modes are also outside the light cone and unable to couple directly with free-space light. However, due to the periodic arrangement, the dispersion of the guided SPP modes are folded back into the light cone by Bragg diffractions, resulting in an upward bended dispersion along k_y at $k_x = 0$ (see **Fig. S4b**). The upward bending can be understood from the intersection of the ω-k_y plane with the first-order diffracted horn-shaped dispersions centered at $k_x = \pm 2\pi/\Lambda$. As shown in **Fig. S4c**, the in-plane k intersecting with the ω-k_y plane at $k_y \neq 0$ (e.g., k_2) is larger than that at $k_y = 0$ (i.e., k_1), resulting in an upward bended dispersion along k_y with $\omega(k_2) > \omega(k_1)$.

For SPPs propagating obliquely to the grooves, the SPP modes behave like coupled waveguide modes. The propagation can be described by the coupled mode theory developed for dielectric and plasmonic waveguide arrays[37–39]. The wavevector k_y of the coupled modes becomes coupled with k_x by

$$k_y(k_x) = \beta_0 + 2C cos(k_x \Lambda), \qquad \text{(S1)}$$

where β_0 is the propagation constant in the waveguide, and C is the coupling coefficient between waveguide modes. **Fig. S5** shows the measured angle-resolved reflectivity spectra at different azimuthal angles (ϕ) for the nanogroove array displayed in the main text. The dispersion along k_x ($\phi = 0^{\circ}$) shows a downward bended curve, while that along k_y ($\phi = 90^{\circ}$) shows an upward bended curve.

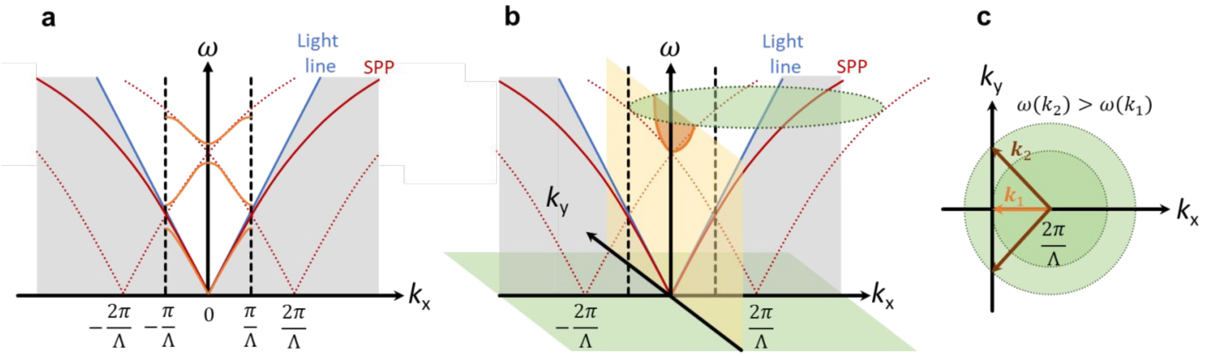

Figure S4 | a, Schematic for SPP dispersion along k_x modified by a periodically arranged nanogrooves with a period of Λ. Due to the periodic structure, the dispersion of SPPs on the flat metal surface (red solid lines) is folded back into the light cone (blue lines, $\omega = kc$) by the Bragg diffraction (red dashed lines) and opens a band gap at Brillouin zone boundaries (black dashed vertical lines). **b**, Schematic for SPP dispersion along k_y at $k_x = 0$ resulting from the intersection of the ω-k_y plane with the first-order diffracted horn-shaped dispersions centered at $k_x = \pm\ 2\pi/\Lambda$. **c**, A top view of two isofrequency contours $\omega(k_1)$ and $\omega(k_2)$ of the first-order diffracted horn-shaped dispersion, which intersect with the ω-k_y plane at k_1 and k_2, respectively.

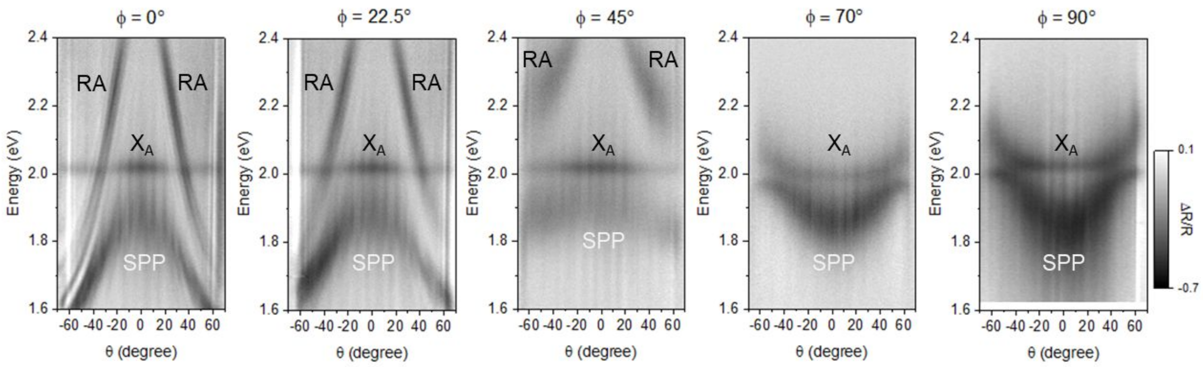

Figure S5 | Dispersions along different in-plane directions (denoted by the azimuthal angle φ) measured by angle-resolved reflection. The measured results clearly manifest the simulated dispersion relation in **Figure 1f**. The absorption of WS_2 A exciton and in-plane diffractive order (i.e., Rayleigh anomalies) were also measured.

3. Fourier-space spectroscopy

The k-space dispersions and PL imaging were measured through Fourier space spectroscopy. **Figure S6a** shows a schematic of the optical setup for measurements of angle-resolved reflectivity spectra, PL spectra and PL images in k-space. A 532 nm laser or a white-light source was selected by a flip mirror (M_1) and sent into an objective lens (NA = 0.95). The reflected white light or PL signals from the sample were collected by the same objective lens and sent to a spectrometer through a tube lens (L_1) and a Fourier lens (L_2). A 550 nm longpass filter was used for PL measurements. For polarization-resolved measurements, a quarter-wave plate (QWP) and a linear polarizer (P) were added to select the circularly-polarized PL components. **Fig. S6b** illustrates the detailed optical paths for the Fourier-space spectroscopy. The k-space spectra and images were obtained by projecting the image on the back focal plane (the Fourier plane) of the objective lens to the entrance slit through L_1 and L_2 in the so-called 4f configuration. The sample was mounted on a rotator, so that the dispersion along any in-plane direction defined by the axis parallel to the entrance slit of the spectrometer can be measured. For k-space PL imaging, the entrance slit was fully opened and the diffraction grating was rotated to zero degree to act as a mirror. The k-space PL imaging was then detected by a CCD camera.

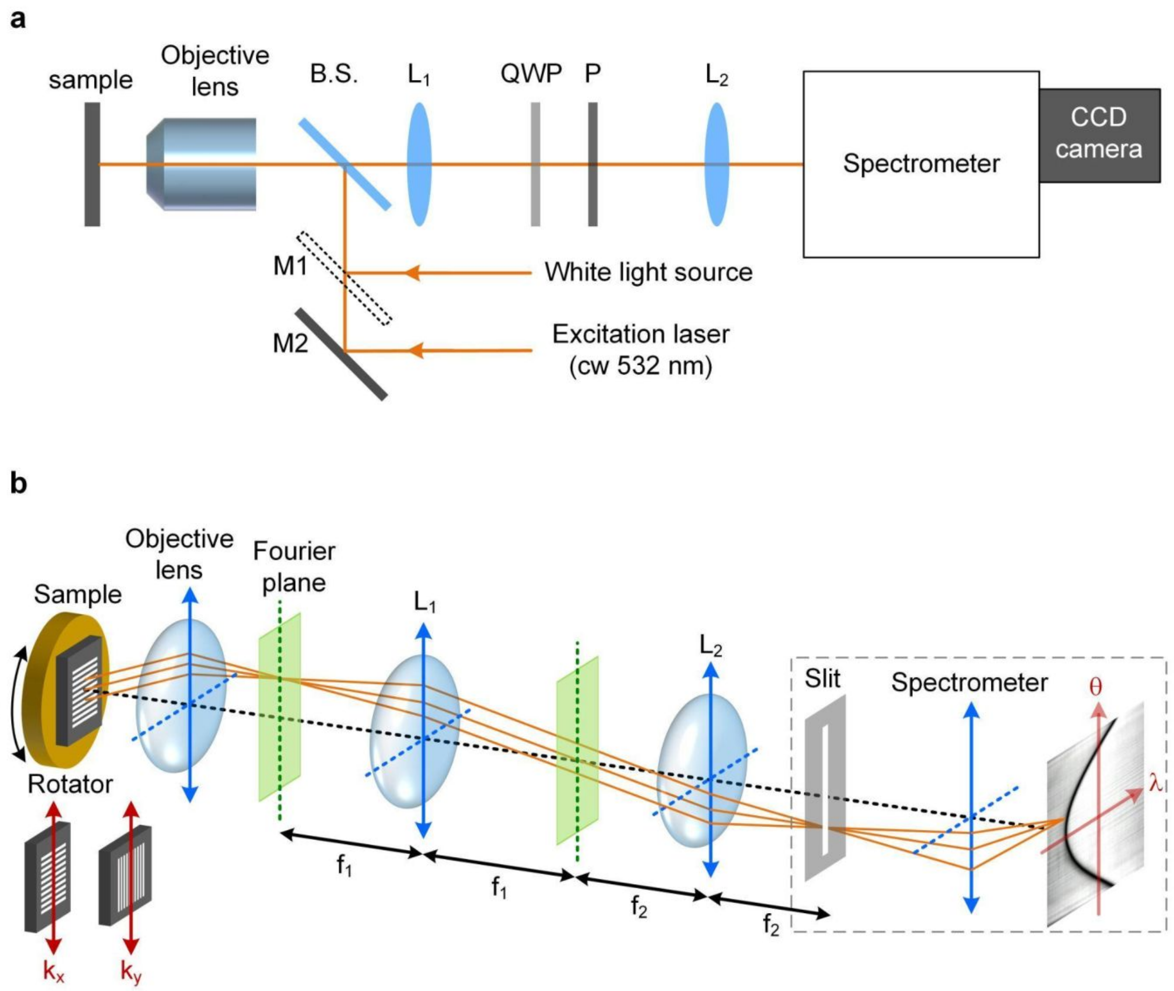

Figure S6 | Schematics of the optical setup (**a**) and the optical paths (**b**) for angle-resolved (Fourier-space) spectroscopy. Samples were mounted on a rotator. The image on the back focal plane (the Fourier plane) of the objective lens was projected on the entrance slit of the spectrometer through lens L_1 and L_2. The CCD camera on the spectrometer recorded the angle (wavelength) in the directions parallel (perpendicular) to the entrance slit.

4. Coupled oscillator model

In the strong coupling regime, a coherent energy exchange occurs between the WS_2 excitons and SPPs with a rate of Rabi frequency. Also, both excitons and SPPs can emit photons spontaneously into the vacuum field and reabsorb these photons, resulting in an incoherent energy exchange without maintaining the phase relationship. **Fig. S7a** shows the FDTD calculated and experimentally measured angle-resolved reflectivity. The strong coupling between X_A of WS_2 and SPPs is evident by the anti-crossing of the dispersions of the upper (UP) and lower (LP) polariton modes.

We analyze the strong coupling of our system by a coupled oscillator model including both the coherent and incoherent couplings between excitons (X) and SPPs. The polariton dispersions can be described by the effective non-Hermitian Hamiltonian:

$$\left[\begin{pmatrix} \tilde{E}_{SPP} & g \\ g & \tilde{E}_X \end{pmatrix} - i\begin{pmatrix} 0 & \gamma_{ic} \\ \gamma_{ic} & 0 \end{pmatrix}\right]\begin{pmatrix} \alpha \\ \beta \end{pmatrix} = E\begin{pmatrix} \alpha \\ \beta \end{pmatrix} \qquad \text{(S2)}$$

where $\tilde{E}_\mathrm{X} = E_\mathrm{X} - i\Gamma_\mathrm{X}/2$ and $\tilde{E}_\mathrm{SPP} = E_\mathrm{SPP} - i\Gamma_\mathrm{SPP}/2$ are the complex resonance energies of the uncoupled monolayer WS_2 excitons and SPPs, respectively. The real parts correspond to resonant energies, while the imaginary parts represent the damping (population decay) by radiative and nonradiative processes (the full width at half maximum linewidth) of excitons and SPPs. The coherent coupling process is described by the coupling strength g between excitons and SPPs. The influence of the incoherent coupling is included by a non-Hermitian cross-damping term γ_{ic}. By solving the eigenvalue problem of **Eq. S2**, we can obtain the dispersion relations of the hybrid exciton-polariton modes. The complex eigenenergies are given by:

$$\tilde{E}_{\pm} = \frac{\tilde{E}_\mathrm{X}+\tilde{E}_\mathrm{SPP}}{2} \pm \sqrt{\left(\frac{\tilde{E}_\mathrm{X}-\tilde{E}_\mathrm{SPP}}{2}\right)^2 + \left(g^2 - \gamma_{ic}^2\right) - 2ig\gamma_{ic}} \qquad \text{(S3)}$$

We extract the dispersions and spectral linewidths of LP and UP modes by fitting the measured angle-resolved reflectivity spectra to a Fano-like line shape[31]. The angle-independent exciton energy and spectral width of WS_2 are taken as 2.03 eV and 30 meV from the measured reflectivity spectrum of WS_2 on flat Ag. As one can see from **Eq. S3**, the two polariton branches exhibit the same linewidth at zero detuning if the cross-damping term vanishes ($\gamma_{ic} = 0$). As $\gamma_{ic} \neq 0$, the linewidths of UP and LP branches always exhibit a difference at zero detuning. By fitting the angle-dependent resonant energies and linewidths of UP and LP branches to the real and imaginary part of **Eq. S3**, we deduced a coupling strength of $g = 59 \pm 2$ meV and a cross-

damping of $\gamma_{ic} = 5 \pm 1$ meV. For the fitted X_A linewidth $\Gamma_X = 39$ meV and the SPP linewidth $\Gamma_{SPP} = 195$ meV, the coupling strength g marginally satisfies the criterion for strong coupling, i.e., $g \gtrsim (\Gamma_X + \Gamma_{SPP})/4$.

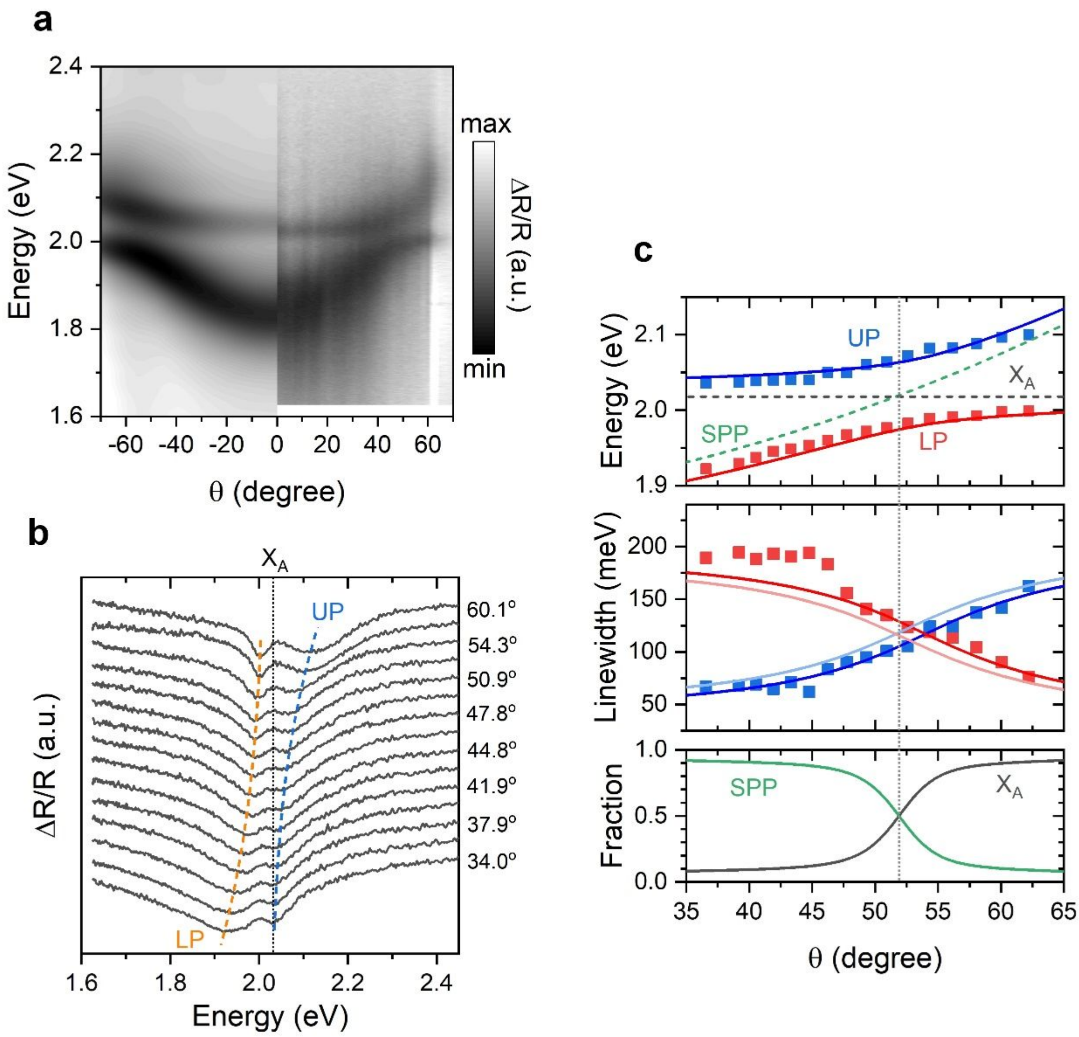

Figure S7 | Coupled oscillator model for exciton-SPP coupling. **a**, Dispersions of excitons and SPPs in the strong coupling regime obtained from measured angle-resolved reflectivity spectra. **b**, The resonant energies (top panel) and linewidths (middle panel) of UP and LP branches and the fractions (bottom panel) of excitons and SPPs of the LP branch as a function of incident angle θ. Blue (UP) and red (LP) symbols are experimental data. The solid lines are fitting curves from the coupled oscillator model. The dashed lines (top panel) are uncoupled dispersions of excitons and SPPs. The light-blue (light-red) solid lines are calculated linewidth of the UP (LP) branch from Eq. S3 without including the incoherent coupling term γ_{ic}. The dimension (w, d, Λ) of the nanogroove array is (50 nm, 80 nm, 400 nm).

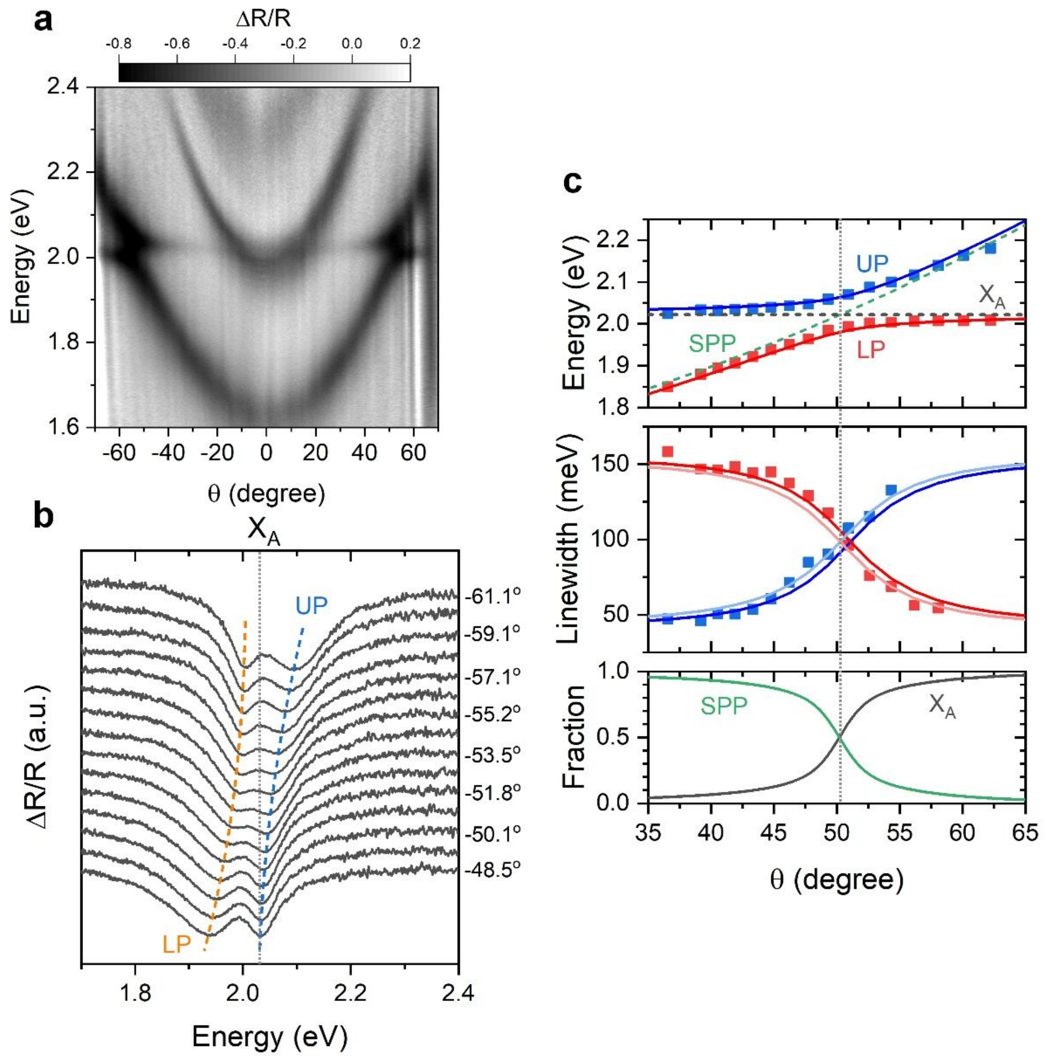

Figure S8 | Coupled oscillator model for exciton-SPP coupling on another nanogroove array. **a**, Dispersions of excitons and SPPs in the strong coupling regime obtained from angle-resolved reflectivity spectra. **b**, The resonant energies (top panel) and linewidths (middle panel) of UP and LP branches and the fractions (bottom panel) of excitons and SPPs of the LP branch as a function of incident angle θ. Blue (UP) and red (LP) symbols are experimental data. The solid lines are fitting curves from the coupled oscillator model. The dashed lines (top panel) are uncoupled dispersions of excitons and SPPs. The light-blue (light-red) solid lines are calculated linewidth of the UP (LP) branch from Eq. S3 without including the incoherent coupling term γ_{ic}. The coupling strength and cross-damping are $g = 50 \pm 2$ meV and $\gamma_{ic} = 2 \pm 1$ meV, respectively. For the fitted X_A linewidth $\Gamma_X = 42$ meV and the SPP linewidth $\Gamma_{SPP} = 155$ meV, the coupling strength g marginally satisfies the criterion for strong coupling, i.e., $g \gtrsim (\Gamma_X + \Gamma_{SPP})/4$. The dimension (w, d, Λ) of the nanogroove array is (60 nm, 60 nm, 600 nm).

5. Additional data for exciton-SPP coupling on other nanogroove arrays

Figure S9 shows the optical characterization of monolayer WS_2 on another nanogroove array (with $w = 60$ nm, $d = 50$ nm and $\Lambda = 500$ nm) different from that presented in the main text. Angle-resolved reflectivity spectra along k_x and k_y are shown in **Fig. S9a,b**. The X_A at 2.03 eV resonates to the SPP_y modes. Clear anti-crossing of the dispersions along ky indicates strong coupling between X_A of WS_2 and the SPP_y of the nanogroove array. **Fig. S9c** shows the PL dispersion along k_y and the unpolarized k-space PL image. The σ+ and σ- circularly-polarized k-space PL images are shown in **Fig. S9d,e**, respectively. **Fig. 9f** shows the corresponding degree of circular polarization (DOP). Real space PL images of σ+ and σ- components of polariton emissions, and the corresponding real-space valley polarization are shown in **Fig. S9g-i**. A separation of σ+ and σ- PL components can be observed.

Figure S10 shows the optical characterization of monolayer WS_2 on another nanogroove array (with $w = 40$ nm, $d = 30$ nm and $\Lambda = 400$ nm). It exhibits coupling between the X_A of WS_2 and the SPP_x of the nanogroove array. Angle-resolved reflectivity spectra along k_x and k_y are shown in **Fig. S10a,b**. The X_A and X_B at 2.03 eV and 2.4 eV coupled to the SPP_x and SPP_y modes, respectively. **Fig. S10c** shows the unpolarized k-space PL image and the PL dispersion along k_x. A PL enhancement is observed when X_A resonates with the SPP_x mode. **Fig. S10d-f** shows the polarization-resolved PL images. The σ^+ and σ^- PL couples to SPPs propagate in opposite directions, and thus out couples to the far field with opposite wavevectors. The degree of circular polarization reaches around ~20% in this case as shown in **Fig. S10f**. The valley polarization contrast was calculated from the normalized quantity $(I_{\sigma+} - I_{\sigma-})/(I_{\sigma+} + I_{\sigma-})$, where $I_{\sigma+}$ and $I_{\sigma-}$ represent the measured PL intensity of left- and right-handed circularly-polarized components, respectively. The polarization-resolved real-space PL images of σ+ and σ- components, and the corresponding DOP are shown in **Fig. S10g-i**.

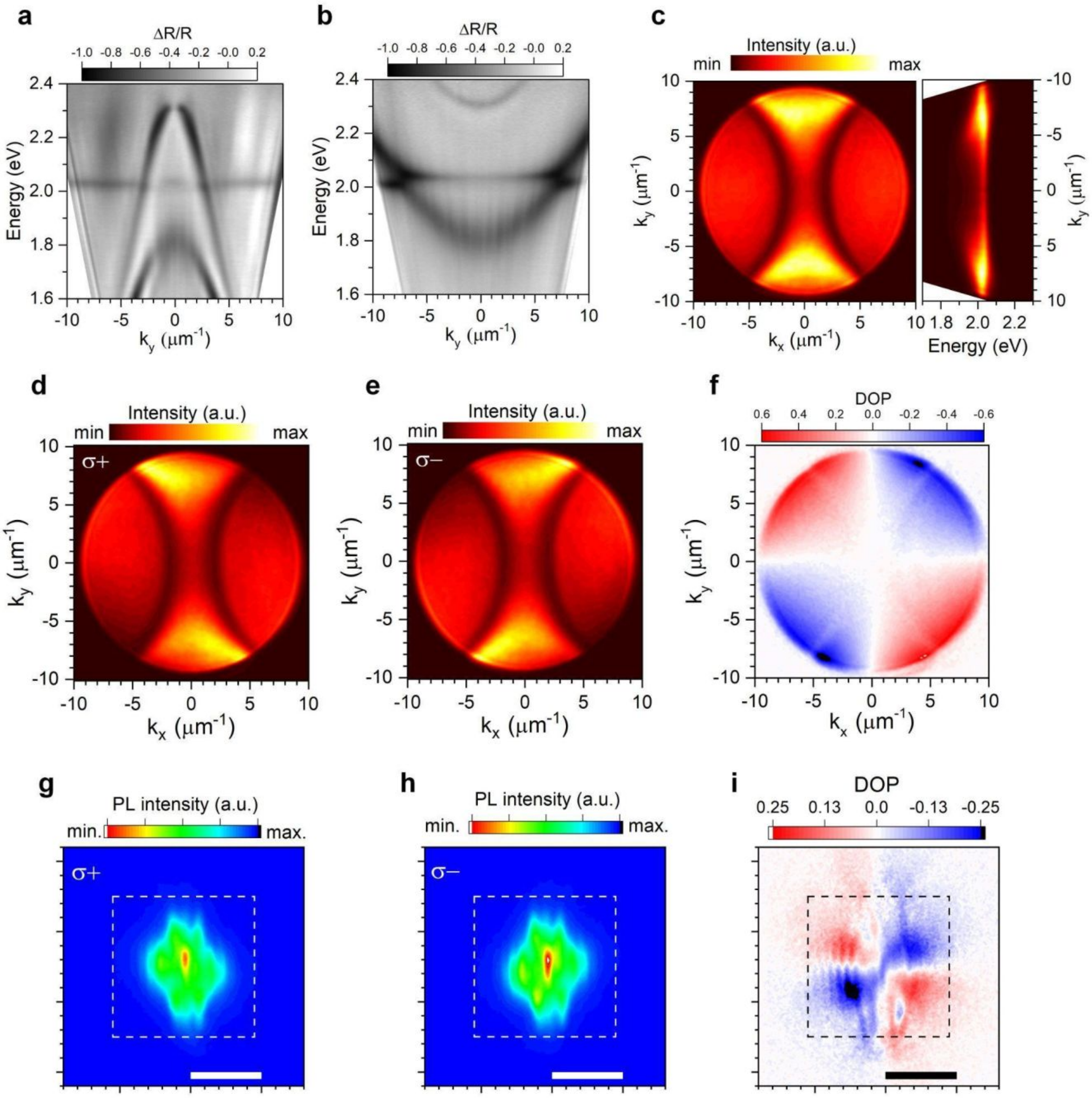

Figure S9 | Exciton-SPP strong coupling in WS_2 on another nanogroove array. **a, b,** Dispersions of excitons and SPPs obtained from angle-resolved reflectivity spectra along k_x (**a**) and k_y (**b**). The A exciton of WS_2 resonates with the SPP_y mode. **c,** The unpolarized k-space PL image and the PL dispersion along k_y. **d-f**, Polarization-resolved k-space PL images of σ^+ (**d**) and σ^- (**e**) PL components, and the corresponding DOP (**f**). **g-i**, Polarization-resolved real-space PL images of σ^+ (**g**) and σ^- (**h**) PL components, and the corresponding DOP (**i**). The dashed box indicates the region of the nanogroove array. Scale bars in **g-i** are 5 μm. The coupling strength and cross-damping of this sample are $g = 46 \pm 2$ meV and $\gamma_{ic} = 6 \pm 1$ meV, respectively. For the fitted X_A linewidth $\Gamma_X = 45$ meV and the SPP linewidth $\Gamma_{SPP} = 175$ meV, the coupling strength g doesn't meet the criterion for strong coupling, i.e., $g < (\Gamma_X + \Gamma_{SPP})/4$.The dimension ($w$, d, Λ) of the nanogroove array is (60 nm, 50 nm, 500 nm).

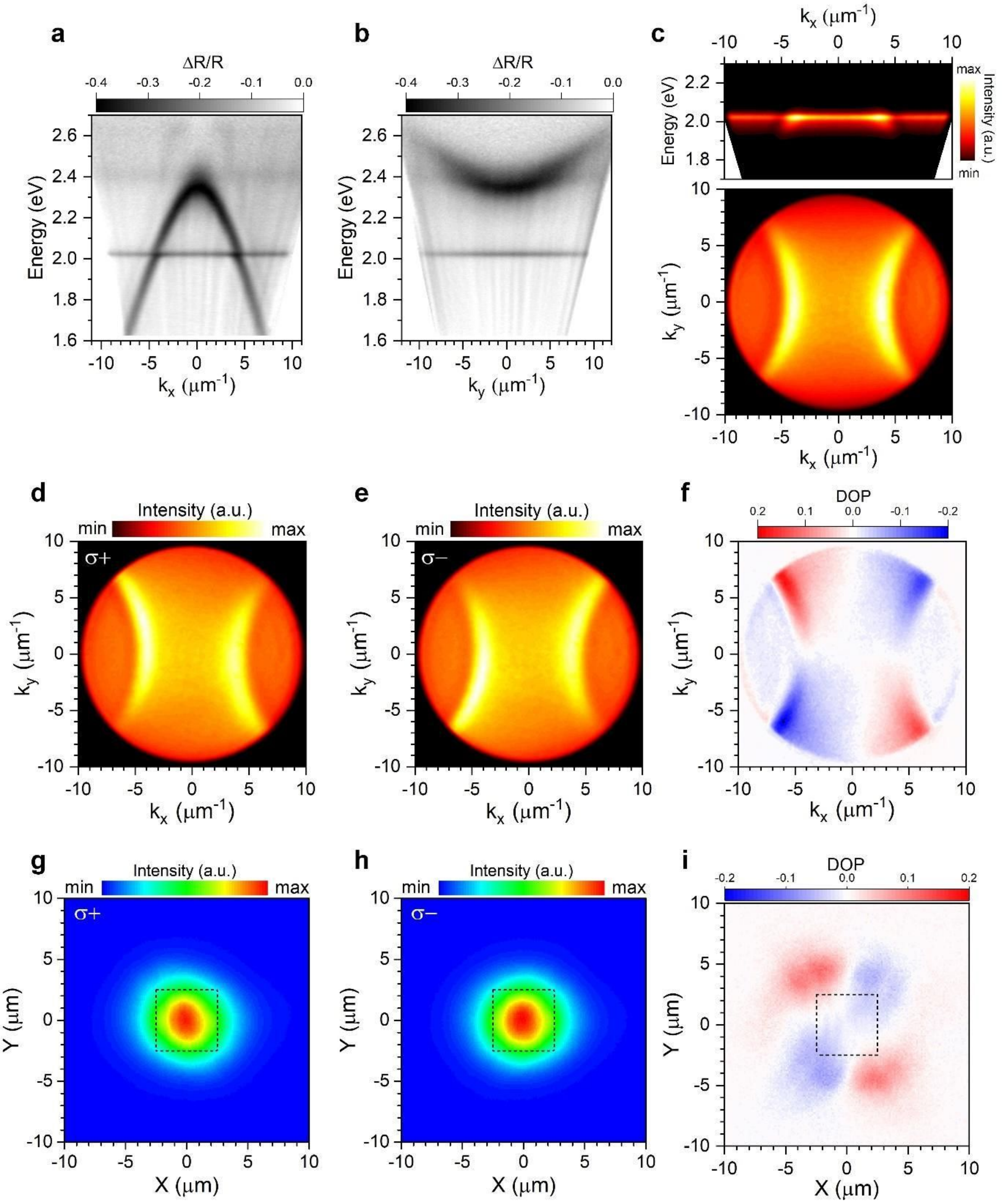

Figure S10 | Exciton coupled with SPP_x in WS_2 on another nanogroove array. **a, b,** Dispersions of excitons and SPPs obtained from angle-resolved reflectivity spectra along k_x (**a**) and k_y (**b**). The A and B excitons of WS_2 resonate with SPP_x and SPP_y mode, respectively. **c,** The unpolarized k-space PL image and the PL dispersion along k_x. **d-f**, Polarization-resolved k-space PL images of σ^+ (**d**) and σ^- (**e**) PL components, and the corresponding DOP (**f**). **g-i**, Polarization-resolved real-space PL images of σ^+ (**g**) and σ^- (**h**) PL components, and the corresponding DOP (**i**). The dashed box indicates the region of the nanogroove array. The dimension (w, d, Λ) of the nanogroove array is (40 nm, 30 nm, 400 nm).

6. Numerical calculations

To visualize the helicity-dependent directional coupling of SPP modes, we use FDTD to simulate the polariton emission by monitoring the far-field radiation of SPP modes excited by circular dipoles. The structure parameters of the simulated metasurface are $w = 50$ nm, $\Lambda =$ 400 nm and tuning $d = 40$-85 nm to achieve exciton-SPP coupling. A 5-nm Al_2O_3 is added on the silver surface. In the simulation, in-plane circular dipoles with $\sigma^{\pm}$ polarization $(p_x \pm ip_y)$ were placed at a distance of 0 nm (5 nm) above the array (silver) surface to excite SPP modes propagating on the *x*-*y* plane. We first record the near-field distributions of the radiated electric-field intensity above the nanogroove array excited by the in-plane circular dipoles. The far-field distributions were then calculated by a projection function to obtain the angular distribution on a hemispherical surface. As illustrated in **Fig. S11a**, right and left circularly-polarized dipoles were placed on top of a nanogroove and at the center of the array (indicated by the red dot). A 2D *x*-*y* electric-field monitor was placed at 1 nm above the array to record the electric-field intensity. Perfectly matched layers were used in all the boundaries in the calculation of far-field radiation. The resultant polarization-dependent electric-field intensity distribution of scattered SPPs in k-space are as shown in **Fig. 3d-f**, with opposite distribution of σ+ and σ- excitation. Simulations of left and right circular dipoles were carried out separately. The electric-field intensities were recorded separately and used to calculate the degree of polarization.

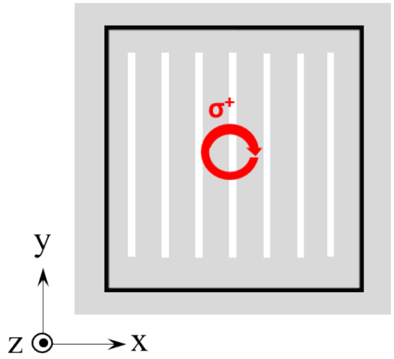

Figure S11 | FDTD calculation of SPP launching by circular dipoles with different helicities. **a**. Schematics of directional coupling of circularly polarized dipoles on nanogrooves.

7. Propagation of valley polariton.

The propagation of valley excitons or coupled valley-polaritons of the hybrided WS2-nanogrooves can be characterized by the near field PL distribution. We discuss the measurement of exciton transport and provide additional real space PL images in this section. The real space PL images were measured by using the optical setup depicted in **Fig. S6**, with the tube lens L_1 removed from the beam path. Pure exciton diffusion was obtained by measuring the PL images of as grown WS_2 on sapphire, and compared to the image of the excitation laser beam spot as shown in **Fig. S12a**. The diffusion length can be obtained by fitting the WS_2 PL intensity distribution by the convolution of the laser profile with an exponential decay function.

The influence of the plasmonic nanogrooves on the transport of TMD excitons can be characterized by comparing the real space PL images of monolayer WS_2 on different substrates. **Fig. S12b** shows the comparison of real space PL images of WS_2 on a flat Ag surface. It can be seen that the PL extent is enlarged significantly, which can be attributed to the launching of SPP waves on the metal surface. As discussed in the main text, the real space PL distribution can be connected to the k-space PL dispersions. **Fig. S12c** shows the real space PL image of WS_2 on the nanogroove array shown in the main text. The PL distribution is elongated along the y direction due to the effect of nanogroove array. The normalized PL intensity profiles of WS_2 on sapphire, on flat Ag surface and on the nanogroove array along the x and y directions are shown in **Fig. S12d-f**. The PL intensity of WS_2 on nanogrooves along the y direction is larger than that on a flat Ag surface.

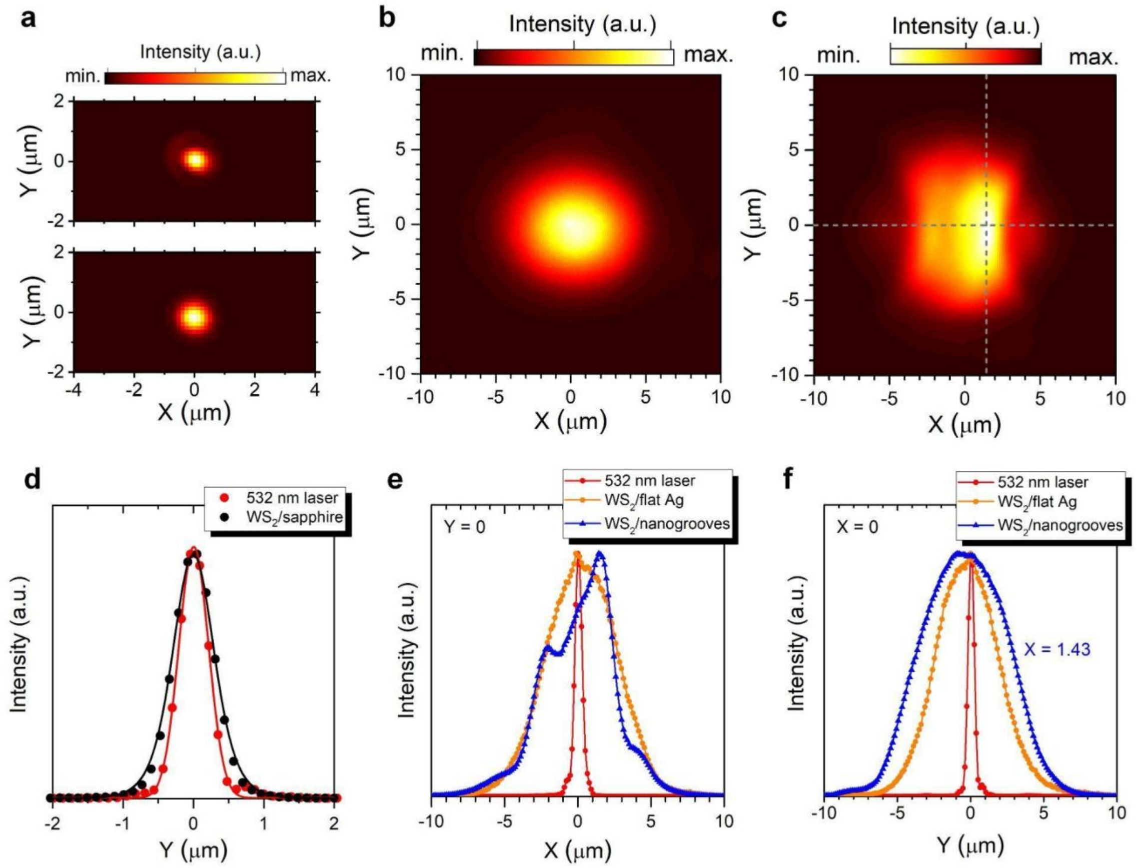

Figure S12 | Real space PL images. **a**, The excitation laser spot image (top panel) and the PL image (bottom panel) of WS_2 on sapphire. **b,c,** The PL images of monolayer WS_2 on flat Ag surface (**b**) and on the nanogroove array (**c**) in main text. **d-f,** The normalized PL intensity profiles of WS_2 on sapphire (**d**) and on the nanogroove array along the x direction at $y = 0$ (**e**) and along the y direction at $x = 1.43$ μm (**f**). The laser intensity profiles are also shown for comparison. The solid lines in (**d**) are fitting curves for the laser profile (Gaussian) and the PL data (convolution of laser profile with an exponential function). The intensity profiles of WS_2 on a flat Ag surface are shown in (**e,f**).

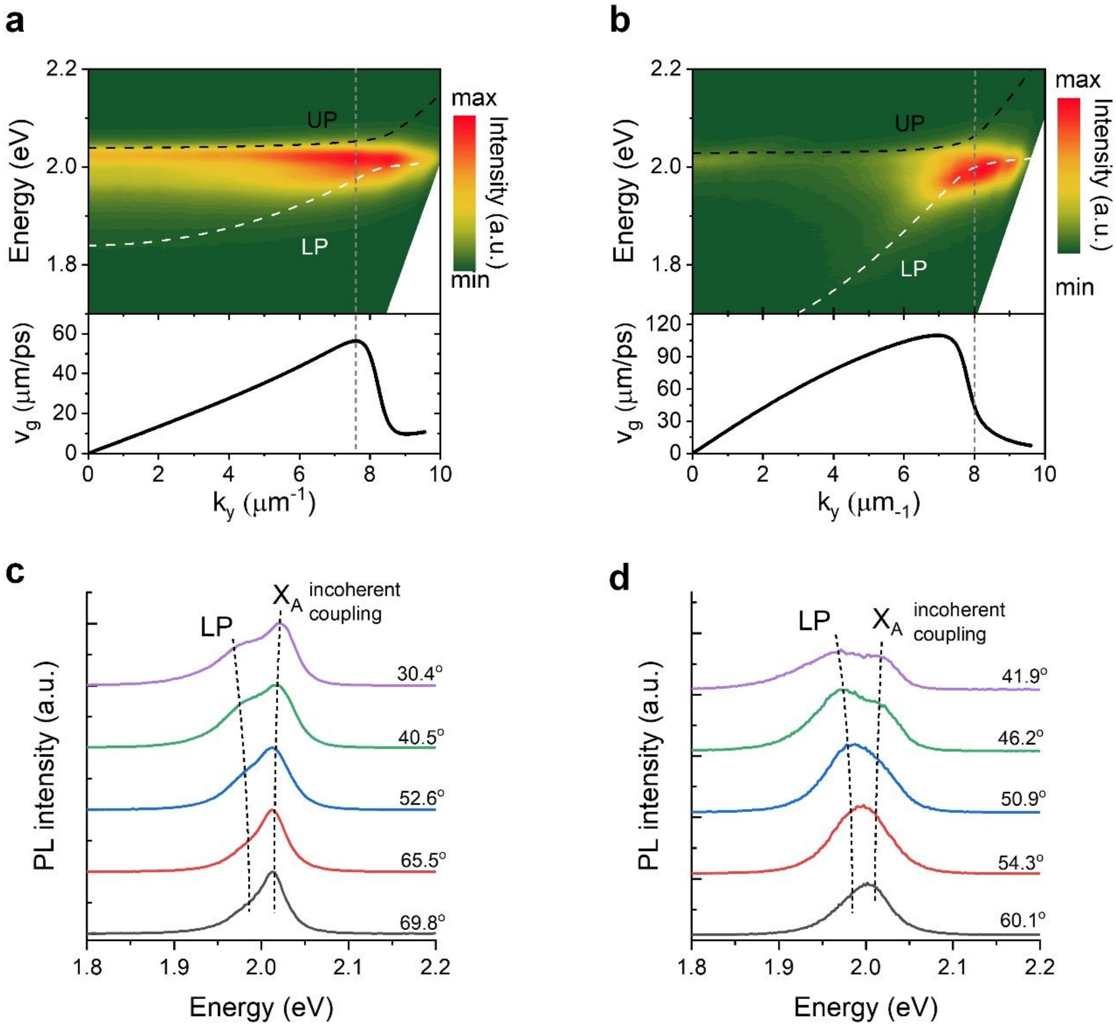

Figure S13 | Group velocity of polariton. a,b, The PL dispersion, the simulated dispersions of UP and LP branches, and the calculated group velocity ($v_g = d\omega/dk_y$) of the LP branch of the coupled WS_2-nanogrooves shown in Fig. S7 (**a**) and Fig. S8 (**b**). **c,d**, The corresponding angle-resolved PL spectra shown in (**a**) and (**b**), respectively.

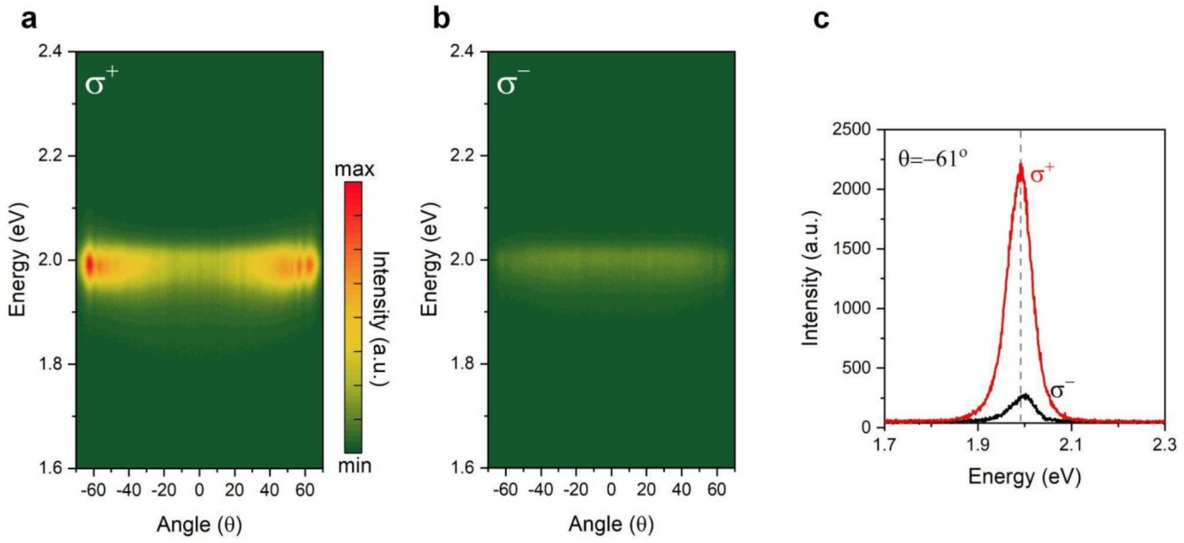

Figure S14 | Spectrally and angle-resolved PL at φ= 110° for σ^+ (**a**) and σ^- (**b**). c, Polarization-resolved PL spectra at θ= -61°. Angles of (θ, φ) are as defined in Fig. 1c in the main text.

References

1. Cheng, F.; Lee, C.-J.; Choi, J.; Wang, C.-Y.; Zhang, Q.; Zhang, H.; Gwo, S.; Chang, W.-H.; Li, X.; Shih, C.-K. Epitaxial Growth of Optically Thick, Single Crystalline Silver Films for Plasmonics. *ACS Appl. Mater. Interfaces* **11**, 3189–3195 (2019).
2. Handbook of Optical Constants of Solids I - III by E. Palik.
3. Phys. Rev. B 90, 205422 (2014).
